# Increased plasma genistein after bariatric surgery could promote remission of NAFLD in patients with obesity

**DOI:** 10.3389/fendo.2022.1024769

**Published:** 2023-01-04

**Authors:** Geng Wang, Yu Wang, Jie Bai, Gang Li, Yang Liu, Shichang Deng, Rui Zhou, Kaixiong Tao, Zefeng Xia

**Affiliations:** ^1^Department of Gastrointestinal Surgery, Union Hospital, Tongji Medical College, Huazhong University of Science and Technology, Wuhan, China; ^2^Department of Gastrointestinal Surgery, Second Affiliated Hospital, Zhejiang University School of Medicine, Hangzhou, Zhejiang, China; ^3^Cancer Center, Union Hospital, Tongji Medical College, Huazhong University of Science and Technology, Wuhan, China

**Keywords:** bariatric surgery, genistein, gut microbiota, non-alcoholic fatty liver disease, obese, sleeve gastrectomy

## Abstract

**Background:**

Bariatric surgery is associated with a positive effect on the progress of non-alcoholic associated fatty liver disease (NAFLD). Although weight loss is the obvious mechanism, there are also weight-independent mechanisms.

**Methods:**

We collected blood samples from 5 patients with obesity before and 3 months after surgery and performed an LC-MS-based untargeted metabolomics test to detect potential systemic changes. We also constructed sleeve gastrectomy (SG) mice models. The plasma, liver and intestine samples were collected and analyzed by qPCR, ELISA and HPLC. Cohousing experiments and feces transplantation experiments were performed on mice to study the effect of gut microbiota. Genistein administration experiments were used to study the in vivo function of the metabolites.

**Results:**

Plasma genistein (GE) was identified to be elevated after surgery. Both clinical data and rodent models suggested that plasma GE is negatively related to the degree of NAFLD. We fed diet-induced obese (DIO) mice with GE, and we found that there was significant remission of NAFLD. Both in vivo and in vitro experiments showed that GE could restrict the inflammation state in the liver and thus relieve NAFLD. Finally, we used co-housing experiments to alter the gut microbiota in mice, and it was identified that sleeve gastrectomy (SG) mice had a special gut microbiota phenotype, which could result in higher plasma GE levels. By feces transplantation experiment (FMT), we found that only feces from the SG mice (and not from other lean mice) could induce higher plasma GE levels.

**Conclusion:**

Our studies showed that SG but not calorie restriction could induce higher plasma GE levels by altering the gut microbiota. This change could promote NAFLD remission. Our study provides new insights into the systemic effects of bariatric surgery. Bariatric surgery could affect remote organs via altered metabolites from the gut microbiota. Our study also identified that additional supplement of GE after surgery could be a therapy for NAFLD.

## Introduction

The prevalence of non-alcoholic fatty liver disease (NAFLD), an obese-related disease, is rising fast and is a growing economic burden all over the world[spice] (1). Previous studies confirmed that NAFLD is typically accompanied by central obesity and metabolic syndrome (MetS) (2). During the progress of NAFLD and non-alcoholic steatohepatitis (NASH), the liver’s capacity to handle the primary metabolic energy substrates is overwhelmed and toxic lipid species accumulate in the liver. Thus, in order to treat NAFLD, we need to control the fatty acid supply and restore the disposal function in the liver. By now, there are many ongoing clinical trials of pharmacotherapies for the treatment of NASH, including CCR2/5 inhibitor, PPARα/δ ligand, FXR ligand/agonist, and ASK-1 inhibitor (1). In addition to drugs, bariatric surgery has been proven to be another powerful therapeutic method for NAFLD and NASH (3–5).

Although many guidelines stated that it is premature to consider bariatric surgery as an option to specially treat NASH (6), clinical data confirmed that surgery is the most effective treatment for weight loss and thus resolved NAFLD and NASH in up to 80% of the patients (7). Notably, some studies suggested that there are weight-independent factors involved. Gut microbiota and bile acid composition may be very important during this process (7). Obesity could decrease microbial diversity and cause a reduction of certain bacterium types (8). Our previous study and other studies showed that this could lead to changes in metabolites (9, 10). The metabolites may enter the circulation system and reach the liver *via* the portal vein. Our study on animal models confirmed that metabolites from the gut microbiota played important roles in NAFLD restriction after sleeve gastrectomy (11). However, the effects and mechanism of the metabolites on NAFLD in patients with obesity remain largely unknown.

In the current study, we collected plasma samples from patients with obesity before and after bariatric surgery. Metabolite sequencing was performed to analyze the differences. We identified genistein (GE) as a special metabolite, as it is increased after bariatric surgery. Since previous studies suggested that genistein is a member of phytoestrogen and could improve insulin resistance, we tried to investigate whether increased genistein after sleeve gastrectomy (SG) plays a role in NAFLD remission.

## Methods

### Human blood specimen and clinical data collection

From June to December 2020, five patients with obesity were included in the study. All the patients had normal levels of fasting blood glucose (FBG) and HbAlc. They all received SG and were safely discharged without any complications. They were followed up for 3 months. Their clinical characters are listed in **Table 1**. Informed consent was obtained from all the patients in accordance with the Declaration of Helsinki and its subsequent amendments. Peripheral blood was collected from patients before surgery and 3 months after SG surgery and centrifuged at 3,000g for 10 min, and the supernatant was collected and stored in a -80°C refrigerator for later use. Laboratory examination was conducted to study the plasma ALT, AST, UA, HDL, and LDL by the Department of Laboratory Medicine. The CT protocol used abdominal CT scan performed using GE (GE 750HD (64), GE Health, Waukesha, Wisconsin, USA). Quality control and image analysis were performed by the same radiologist. A CT diagnosis of hepatic steatosis was made by measuring liver attenuation (LA) in Hounsfield units (HU). Lower LA was related to higher lipid accumulation (12), and CT values ≤40 HU were considered as NAFLD (13, 14).

**Table 1:** Clinical baselines and characteristics before and 3 months after SG.

Patient number	1	2	3	4	5
Age	32	23	31	35	27
Gender	Female	Female	Female	Female	Female
Baseline BMI	40.6	43.7	36.4	38.1	44.1
Postsurgical BMI	31.7	31.2	26.5	29.1	36.4
Type 2 DM	No	No	No	No	No
Hypertension	No	No	No	Yes	No
NAFLD	Yes	Yes	Yes	Yes	Yes
Baseline ALT (U/L)	189	153	167	59	172
Postsurgical ALT (U/L)	87	69	64	53	102
Baseline AST (U/L)	78	45	63	45	83
Postsurgical AST (U/L)	36	36	35	32	70
Baseline UA (μmol/L)	530	438	462	403	515
Postsurgical UA (μmol/L)	512	422	477	371	428
Baseline LDL (mmol/L)	2.97	3.53	5.53	2.79	4.26
Postsurgical LDL (mmol/L)	2.72	3.26	3.97	2.53	3.45
Baseline HDL (mmol/L)	0.71	0.84	0.75	1.04	0.67
Postsurgical HDL (mmol/L)	0.89	1.13	1.01	1.27	0.94

### Untargeted metabolomics

Untargeted metabolomic analysis was performed using LC-MS/MS technology, and high-resolution mass spectrometer Q Exactive (Thermo Fisher Scientific, USA) was used to collect data in both positive and negative ion modes to improve metabolite coverage. Compound Discoverer 3.0 (Thermo Fisher Scientific, USA) software was used for LC-MS/MS data processing, mainly for peak extraction, peak alignment, and compound identification. The self-developed metabolomics R software package metaX and the metabolome information analysis process were used for data preprocessing, statistical analysis, and metabolite classification annotation and functional annotation. Dimensionality reduction of multivariate raw data was done through PCA (principal component analysis), in order to analyze the grouping, trend (similarity and difference between sample groups and between groups), and outliers of the observed variables in the data set value (whether there are abnormal samples). PLS-DA (partial least squares method-discriminant analysis) was performed to modulate the VIP value of the first two principal components, combined with the fold change obtained by univariate analysis and T test (Student t test) to screen differential metabolites.

### Metabolic assays

Mouse liver metabolic assays were performed as previously reported (15, 16). Briefly, blood was collected from mice through the inner canthus after mice had fasted for 6 h. The serum was separated and stored at -80°C before use. Aspartate aminotransferase (AST) triglyceride (TG) in the liver was measured using commercial kits (Abcam, USA) on a fully automatic biochemical analyzer (Hitachi 917, Japan). The concentrations of GE were tested by the high-performance liquid chromatography (HPLC) system (Thermo Fisher, USA). After centrifugation at 17,000×*g* for 15 min at 4°C, the protein was then precipitated by adding 100 mM HCl and centrifuging at 17,000×*g* for another 15 min at 4°C. The remaining supernatant was injected onto a 250 mm ACE C-18 column (Hichrom, UK), and genistein was quantified using 210 nm UV detection. A mobile phase containing 2.5% acetonitrile and 0.1% phosphoric acid (ultra-pure electrochemical HPLC grade) was used for HPLC separation at a flow rate of 1.0 ml/min. Genistein was quantified by comparison with a standard range of pure Genistein (0.50 to 500 mM). All procedures were performed according to the manufacturer’s instructions.

### Animals and diet

The IACUC number for animal ethics approval is 2468. All animal studies were approved by the Institutional Animal Care and Use Committee of Tongji Medicine College. All applicable institutional guidelines for the care and use of animals were followed. Six-week-old female C57BL/6J mice were purchased from Beijing HFK Bioscience Co., Ltd., and raised in specific pathogen-free (SPF) conditions in the Tongji Medicine School Animal Center. The mice were housed at 22 ± 2°C on a 12-h light cycle. For the diet-induced obesity (DIO) model, the mice were fed a high-fat diet (HFD) (H10060, 60% fat, 20% protein, 20% carbohydrate, Beijing HFK Bioscience Co., Ltd.) for 10 weeks to induce obesity before SG surgery (17).

### SG and postoperative care

SG and postoperational care were performed as previously described (18). Briefly, all the operations were carried out in SPF conditions, and the mice were fasted overnight before surgery. Surgeries were carried out under 1% pentobarbital sodium (80 mg/kg). For SG, the abdominal cavity is opened at the position 2–3 mm below the gastroesophageal junction, ophthalmic scissors are used to start from the left side of the stomach (greater curvature), 80% of the stomach tissue along the great curvature of the stomach is removed, and the fundus is completely removed. Then, the remaining approximately 20% of the stomach is sutured and put back into the abdominal cavity.

For a 4-day perioperative period, mice were provided a liquid diet Osmolite (Abbott Laboratories, Columbus, OH). Osmolite contains approximately 29% of calories from lipid and 54% of calories from hydrolyzable carbohydrate (mostly sugars). After 4 days, all mice returned to a normal diet.

For the DIO-sham group, we also opened the abdominal cavity and, after isolating the gastric tissue and esophagus, we kept the exposure time the same as that of the SG group. Then, we closed the abdominal wall. For the pair-weight group, we frequently recorded the weight of the mice and, by restricting food intake, we kept these mice at similar weight levels compared with the SG mice. Normal-diet mice received a normal diet all the time and served as a control group.

### Analysis of the abundance of GE in serum and other organs

To analyze whether SG could affect the abundance of GE in different parts of the body, we set four groups. There are five mice in each group: the DIO-SG group, the DIO-sham group, the pair-weight group, and the normal diet group. They are age matched. Their body weight and blood samples were collected every week. 6 weeks after surgery, the mice were sacrificed and the intestine, liver, and serum were collected for investigation by qPCR, ELISA, and HPLC.

### Cohousing experiments and feces transplantation experiments

For cohousing experiments, we set two groups. In each group, there are six mice. All the mice were female to avoid fighting in one cage. In the SG group, six DIO mice received SG and they were raised for 6 weeks after surgery. In the SG cohousing group, another six DIO mice also received SG but they were divided into two cages and in each cage the three SG mice were cohoused with three DIO mice. They were also cohoused for 6 weeks.

For the fecal microbiota transplantation (FMT) experiment, we followed the procedures reported previously (19). Briefly, about 0.1 g fresh feces from the donor mice was collected in the 1 ml EP tube. Phosphate-buffered saline (PBS) was added to dilute the stool to 1 ml. The mix was homogenized and filtered. The liquid was then centrifuged at 500g for 5 min at 4°C to remove the large particles. Then, the suspended particles were used for oral gavage immediately. We used 200 μl for the gavage once a day for 2 weeks. Notably, before oral gavage, the recipient mice received antibiotics administration (2000 U/l penicillin + 2 mg/ml streptomycin diluted in drinking water) for 7 days (20).

There are three groups of recipient mice. All the mice were 6-week-old mice fed with a normal diet. In the SG-FMT group, the mice received feces from SG mice (1 month after SG surgery). In the DIO-FMT group, the mice received feces from DIO mice. Moreover, in the pair-weight FMT group, the mice received feces from pair-weight control mice. The serum was collected every week, and the intestine and liver were collected after the mice were sacrificed. The feces from different groups were collected and sent for 16S rRNA gene sequencing using the MO BIO PowerSoil Kit (MO BIO). Principal component analysis (PCA) was performed to confirm the changes of the gut microbiota after cohousing or FMT treatment. Details are shown in **Supplementary Figure 1**.

### GE administration experiments

For GE administration experiments, there are three groups and in each group there are five mice. In the DIO group, the mice received HFD as previously mentioned. In the control group, there are age-matched mice which received a normal diet and had a normal body weight. In the GE group, there were DIO mice fed with HFD containing GE (HFD plus 0.2% GE diet) for 8 weeks[spice]16]. GE was provided by LC Laboratories, USA. Purity was >99%.

For SG+GE experiments, we aimed to study the effects of supplement of GE after SG. There are five mice in each group. The SG group is the DIO mice which underwent SG and fed with HDF. The SG+GE group received the same procedure but was fed with HFD+0.2%GE. After 6 weeks, they were sacrificed and analyzed.

### Mouse sample collection

The mice were sacrificed 6 weeks after the SG operation by CO_2_ anesthesia as previously reported (21). The blood was collected and centrifuged at 4,000g for 15 min to obtain serum. The liver was quickly resected, rinsed with ice-cold PBS, and quickly put in liquid nitrogen. Cecal contents were gently squeezed out of the excised cecum into cold cryotubes and frozen rapidly. Serum, liver, and cecal content samples were stored at -80°C until further analysis.

### HE staining

The liver samples were collected and were fixed in buffered formalin (4%) overnight and embedded in paraffin. Five-micrometer-thick serial sections were prepared from paraffin-embedded tissues and stained with hematoxylin and eosin (H&E) to observe the distribution of lipid accumulation.

### Immunohistochemistry

The tissue sections were deparaffinized and hydrated in water. Sections were incubated for 5–10 min at 37°C to retrieve mycobacterium antigens. Then, they were rinsed thoroughly in Tris-buffered saline and proceeded with the immunostaining procedure. The slides were washed two times for 5 min in TBS plus 0.025% Triton X-100 (Sigma-Aldrich) and then blocked with 1.00% BSA (Sigma-Aldrich) in TBS for 2 h at room temperature. Then, the Rat mAb F4/80 (Abcam, ab15694) was diluted to the manufacturer’s recommendations (1/200) and the sections were incubated overnight at 4°C. After washing two times for 5 min TBS plus 0.025% Triton X-100, the slides were incubated in 0.30% H_2_O_2_ (Sigma-Aldrich) in TBS for 15 min. After two times washing in TBS buffer, secondary antibody goat anti-rat (HRP; Abcam) was diluted in TBS with 1.00% BSA (1/200), applied to slides, and incubated for 1 h at room temperature. The samples were rinsed with buffer TBS plus 0.025% Triton X-100, three times for 10 min, and then 3,3-diamino-benzidine (DAB; Sigma-Aldrich) chromogen was used for 10 min at room temperature. DAB produced a brown precipitate (where the secondary HRP antibody binds to the primary) that was insoluble in alcohol, xylene, and other organic solvents most commonly used in the laboratories. The slides were rinsed in running tap water for 10 min and stained with nuclear counterstained Mayer’s hematoxylin (Sigma-Aldrich) for better visualization of the tissue morphology. Dehydration, clearance, and mounting using a compatible mounting medium were carried out.

### ELISA

The concentrations of TNF-α, IL-1β, and MCP-1 in the serum, liver, and cecum of human were examined by ELISA kits (IL-1β: MLB00C, R&D; TNFα: MTA00B, R&D; MCP-1: MJE00B, R&D). All procedures were performed according to the manufacturer’s instructions.

### RNA extraction and RT-qPCR

Total RNA extraction was performed using the miRNeasy Micro Kit according to the manufacturer’s instructions. Briefly, 500 µl of Qiazol solution (a buffer containing guanidinium thiocyanate, GITC) was used to lyse the tissue. 100 µl of chloroform was added. The mixture was vortexed and centrifuged at 12,000 rpm for 15 min at 4°C. The supernatant was obtained, and 1.5 volume of 100% ethanol was added. The mixture was centrifuged through an extraction column (Qiagen S.A.). The column was washed with RWT buffer and REP buffer, respectively. RNA was eluted in 20 µl of RNase-free water and quantified using NanoDrop. RNA samples were stored at –80°C prior to RT-PCR. Complementary DNA (cDNA) was synthesized from total RNA using PrimeScript™ RT Master Mix Kit (Takara). For RT reactions, 100–500 ng of RNA extract was used. The cDNA specimens were stored at −20°C until PCR. Quantitative real-time PCR was performed using SYBR Green with an ABI StepOnePlus system (Life Technologies, Carlsbad, CA, USA). The primers used are listed in **Table 2**. All the gene expression levels were normalized to the GAPDH levels. The relative quantification of mRNA was calculated by a comparative Ct method (2^−Δct^).

**Table 2:** Sequences of the primers.

Gene name	Primers
*Tnfa*-F	CTGGATGTCAATCAACAATGGGA
*Tnfa*-R	ACTAGGGTGTGAGTGTTTTCTGT
*IL-1β*-F	GAAATGCCACCTTTTGACAGTG
*IL-1β*-R	TGGATGCTCTCATCAGGACAG
*Mcp-1*-F	TTAAAAACCTGGATCGGAACCAA
*Mcp-1*-R	GCATTAGCTTCAGATTTACGGGT
*Gapdh*-F	AGGTCGGTGTGAACGGATTTG
*Gapdh*-R	TGTAGACCATGTAGTTGAGGTCA

### Cell culture and treatment

Cells of murine macrophage RAW264.7 were seeded into 12-well plates at a density of 4 × 10^5^ cells/ml and cultured at 37°C with 5% CO_2_. The culture medium was DMEM containing 10% FBS with 1% penicillin/streptomycin. Cells were pretreated with different concentrations of genistein for 4 h, and then, 300 mM palmitate was added. After 18 h, 10 ng/ml LPS was added to the medium. The cells were then incubated for an additional 6 h. Bone marrow-derived macrophages (BMDMs) were isolated from the femur of wild-type C57/BL6 mice. The isolated progenitor cells were resuspended in RPMI-1640 medium supplemented with 10 ng/ml macrophage colony-stimulating factor (M-CSF) (Invitrogen), seeded in polystyrene dishes, and incubated for 3 days at 37°C and 5% CO_2_ in a humidified incubator. The culture medium was replenished on day 3, and the cells were incubated for an additional 4 days. At the end of the 7-day culture period, >95% of the cells were positive for macrophage markers F4/80 and CD11b.

### Macrophage migration assay

BMDM migration was investigated in Transwell cell culture chambers with polycarbonate membranes (8-mm pore size, Corning Costar, Corning, NY, USA). Cells were added to the upper chamber, and either vehicle or different concentrations of genistein were added to both the upper and lower chambers. After 4 h, 20 ng/ml MCP-1 (Invitrogen) was added to the bottom chamber. After 24 h, cells remaining on the upper side of the membrane were scraped off with a cotton swab. The migrated cells in the bottom chamber were fixed with methanol for 15 min, stained with 0.1% crystal violet for 30 min, and counted under a microscope. Three replicate wells were analyzed in each experiment, with cells counted in 15 randomly chosen fields of view per well.

### Statistical analysis

Data were analyzed with SPSS 21.0 (IBM Corp., USA). Continuous variables were reported as means ± standard deviation. Mann–Whitney U test or Wilcoxon test was used to compare between-group differences. Differences among three or more groups were compared by analysis of the ANOVA test. Spearman correlation analysis was used to analyze the relationship between CT values and serum GE levels. Bar plots were generated with GraphPad Prism 8.0 (GraphPad Software, San Diego, USA). *p* values <0.05 indicated statistical significance. *p < 0.05, **p < 0.01, ***p < 0.001.

## Results

### NAFLD is relieved after SG in patients with obesity

**Figure 1:**
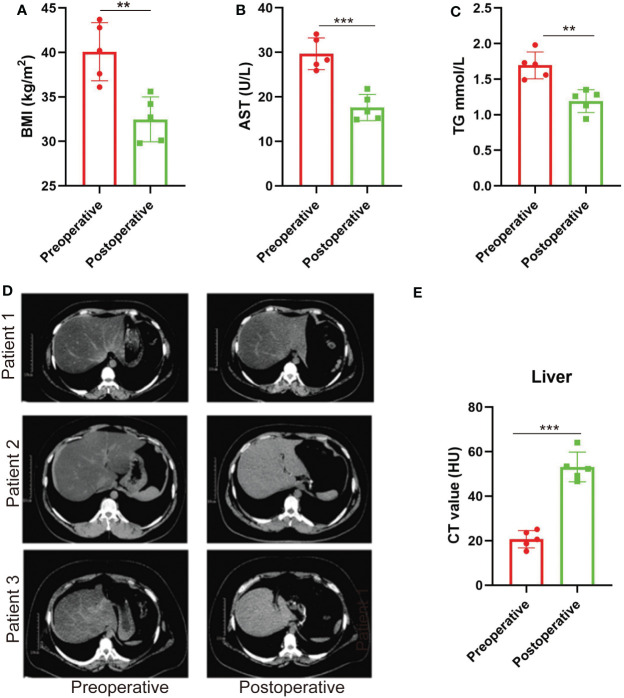
NAFLD is relieved after SG in patients with obesity. **(A–C)** BMI values, plasma AST, and TG before and 3 months after SG on patients with obesity. n = 5. **(D)** CT scan images of the liver in patients with obesity before and 3 months after SG surgery (three out of the five patients). **(E)** CT values of the liver before and 3 months after SG surgery on patients with obesity. n = 5. Wilcoxon test was used to compare the data before and after surgery. Data are presented as mean ± SEM, **p < 0.01, ***p < 0.001.

Clinical baselines and characteristics of the five patients included in the study are listed in **Table 1**. 3 months after SG, the patients with obesity had significantly decreased BMI (**Figure 1A**). Liver function was also improved in terms of the levels of aspartate aminotransferase (AST) and triglyceride (TG) (**Figures 1B, C**). There was also significant remission of NAFLD evaluated by the CT value (**Figures 1D, E**).

### Plasma and local levels of genistein are increased after SG in patients with obesity

**Figure 2:**
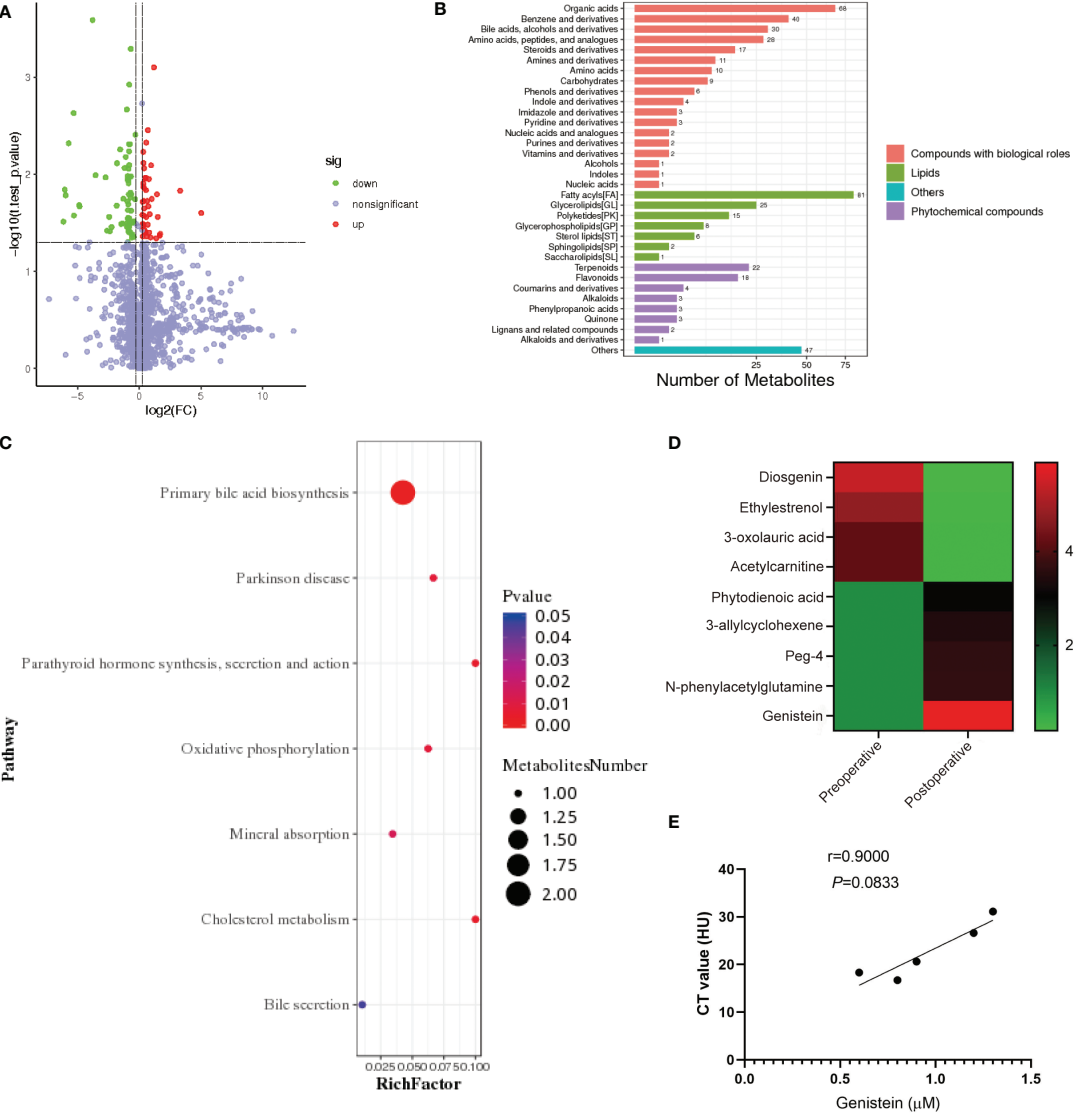
Plasma and local levels of genistein are increased after SG in patients with obesity. **(A)** Metabolite volcano map before and after SG in patients with obesity. **(B)** KEGG shows the affection of SG on metabolism in patients with obesity. **(C)** Analysis of the effect of SG on metabolism in patients with obesity. **(D)** Heatmap of relevant differential metabolites before and after SG. **(E)** Correlation analysis of the NAFLD degree and genistein abundance in patients with obesity. Spearman correlation was performed to study the relationship. Data are presented as mean ± SEM, **p < 0.01, ***p< 0.001.

The blood samples from patients with obesity before surgery and 3 months after surgery were collected and sent for untargeted metabolomic analysis (**Figures 2A–C**). We found that genistein (GE), a member of soy isoflavones, was significantly increased after SG (**Figure 2D**). In patients with obesity, the plasma levels of GE tended to be negatively related to the NAFLD degree (evaluated by CT values), but there was no significant difference due to the limited samples (p = 0.0833) (**Figure 2E**).

### Abundance of genistein is decreased in mice with obesity and could be restored by SG

**Figure 3:**
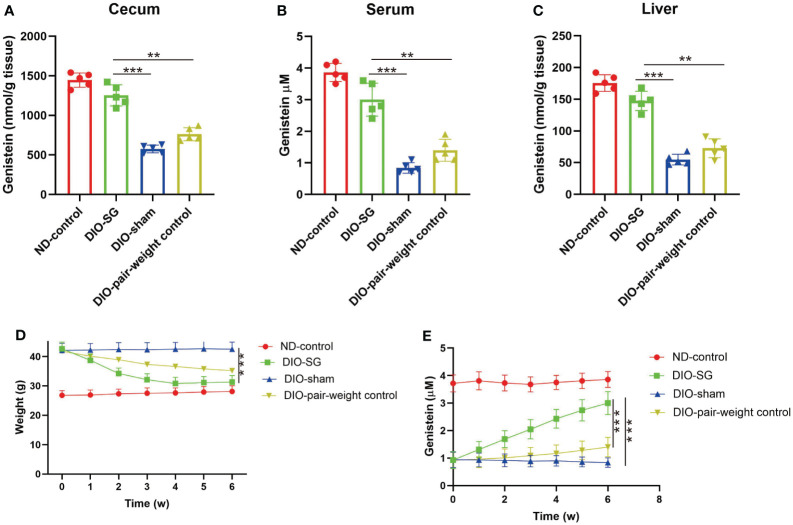
Abundance of genistein is decreased in mice with obesity and could be restored by SG. **(A–C)** The concentration of genistein in the cecum, serum, and liver in different groups. n = 5 in each group. **(D)** The changes of body weight in each group. **(E)** The changes of plasma levels of GE in each group. The details of the group were introduced in the “Methods” part. ANOVA test was used to compare the differences among the groups. Data are presented as mean ± SEM, *p < 0.05.

We further tested this finding in rodent models. We found that in the cecum, liver, and serum, GE is decreased in diet-induced obese (DIO) mice. Moreover, after SG, the GE levels were recovered (**Figures 3A–C**). However, although there is similar weight loss in the pair-weight control group (**Figure 3D**), this group did not have significantly increased plasma GE levels compared with the SG group (**Figure 3E**).

### GE could relieve NAFLD in mice with obese and better relief NAFLD after SG

**Figure 4:**
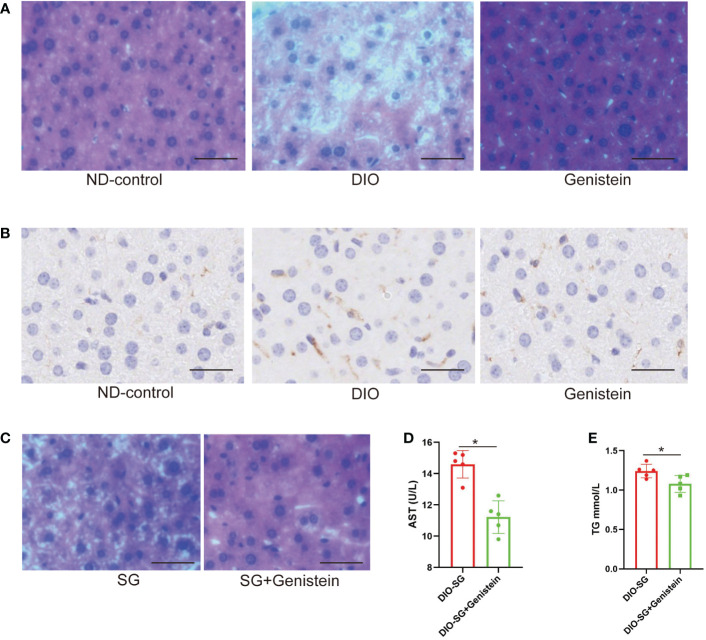
Genistein could relieve NAFLD and decrease the inflammation status in the liver. **(A, B)** The infiltration of macrophages (marked by F4/80) and lipid accumulation were detected by immunohistochemistry in the liver tissue of mice from different age-matched groups. ND-control: normal mice fed with a normal diet. DIO: diet-induced obese mice. DIO+GE: DIO mice fed with HFD containing 2% GE for 8 weeks. Scale bar = 50 μm. **(C)**. HE staining of livers from SG and SG+ GE mice. Scale bar = 50 μm **(D, E)**. Serum AST and TG concentrations in SG and SG + GE mice. The DIO mice received and were fed with HFD (the SG group) or HFD+0.2% GE (the SG+GE group) for 8 weeks. n = 5 in each group. Mann–Whitney U test was used to analyze the differences between the two groups. Data are presented as mean ± SEM, *p < 0.05. The P value in the image used in Figures 4E is equal to 0.0833, less than 0.05.

By feeding DIO mice with GE, we found that NAFLD was relieved by decreased infiltration of macrophages and fewer lipid accumulation (shown by HE staining, **Figures 4A, B**). Interestingly, by adding GE to the diet after SG, the mice had better NAFLD relief compared with those without GE supplement (**Figure 4C**). Additional GE after surgery also contributed to better liver function in terms of serum AST and TG (**Figures 4D, E**).

### GE could decrease inflammation in the liver in mice with NAFLD

**Figure 5:**
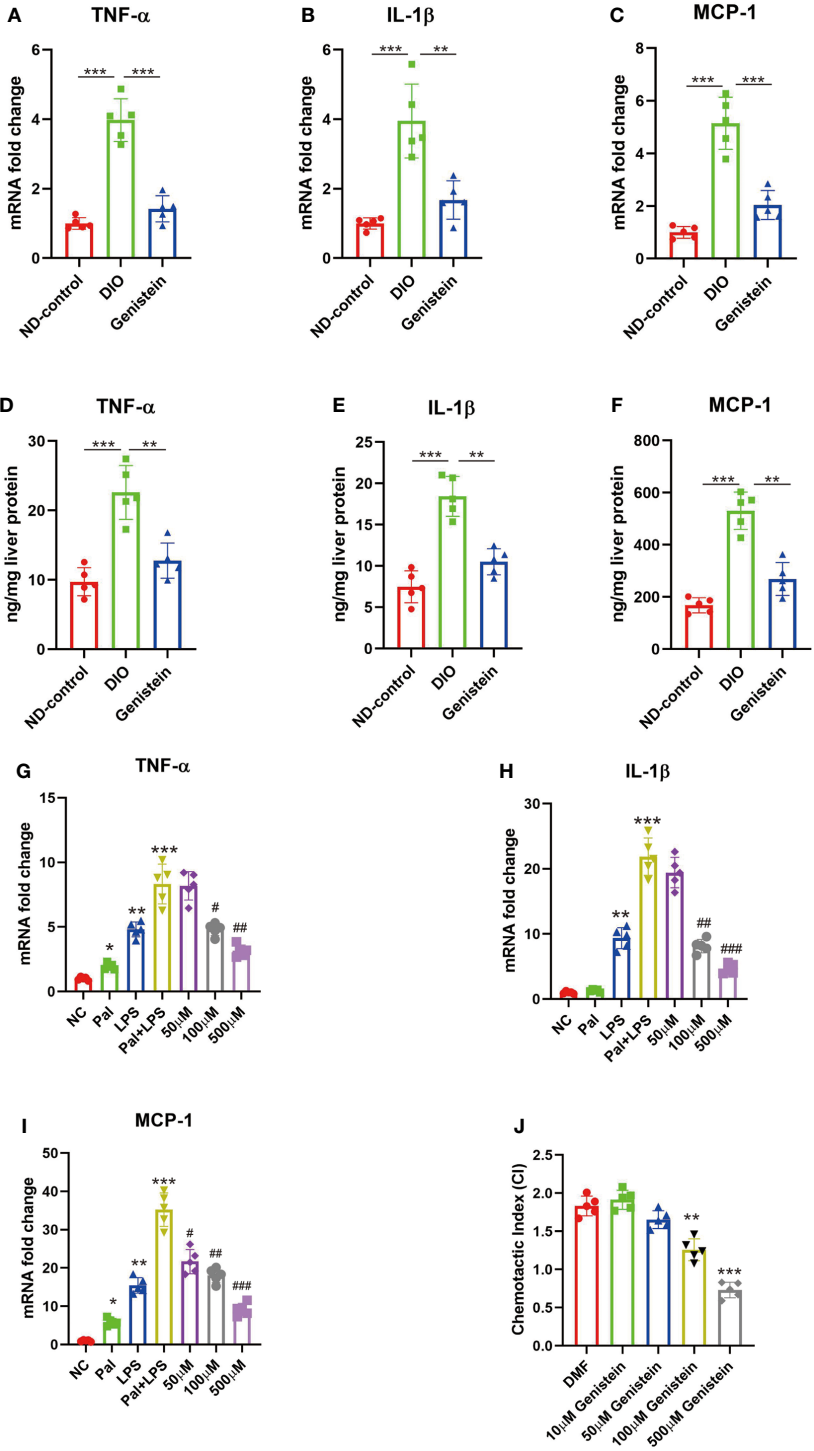
Genistein could reduce pro-inflammatory cytokine production and chemotactic migration in macrophages in the liver. **(A–C)** The mRNA levels of TNF-α, IL-1β, and MCP-1 in the liver from different groups, tested by qPCR. **(D–F)** The protein levels of levels of TNF-α, IL-1β, and MCP-1 in the liver from different groups, tested by ELISA. The age-matched female mice received 6 weeks of normal diet (Control), HFD diet (HFD), and GE diet (HFD+0.2%GE). n = 5 in each group. **(G–I)** RAW 264.7 cells were stimulated with palmitic acid (Pal) followed by LPS, with or without addition of varying doses of genistein. The negative control (NC) group was only treated with the vehicle, dimethylformamide (DMF). Changes in the expression of TNF-a, IL-1β, and MCP-1 were determined using qPCR. **(J)** Genistein inhibits BMDM migration toward MCP-1. BMDMs were incubated with MCP-1 in a Transwell with vehicle (DMF) or different doses of genistein. The chemotactic index (CI) for a treatment condition was calculated as the ratio of average number of migrated cells in the treatment group relative to the control group (incubated in medium only). ANOVA test was used to compare the differences among the groups. Data are presented as mean ± SEM, * stands for p < 0.05, ** stands for p < 0.01 and *** stands for p < 0.001, compared with the negative control (NC) group. For G-I, # stands for the GE stimulation group compared with the Pal+LPS stimulation group, #p < 0.05, ##p < 0.01, ###p < 0.001.

In mice with obesity, there is increased inflammation in the liver and administration of GE could restrict inflammation (**Figures 5A–C** for mRNA levels and **Figures 5D–F** for protein levels). Since *in vivo* experiments suggested that GE could restrict inflammation in the liver, we used *in vitro* experiments to study the effect of GE stimulation on *in vitro* cultured macrophages. The results suggested that GE could inhibit TNF-α, IL-1β, and MCP-1 secretion (**Figures 5G–I**). It could also restrict macrophage migration (**Figure 5J**).

### Transplantation of feces from SG mice could induce GE abundance in recipient mice

**Figure 6:**
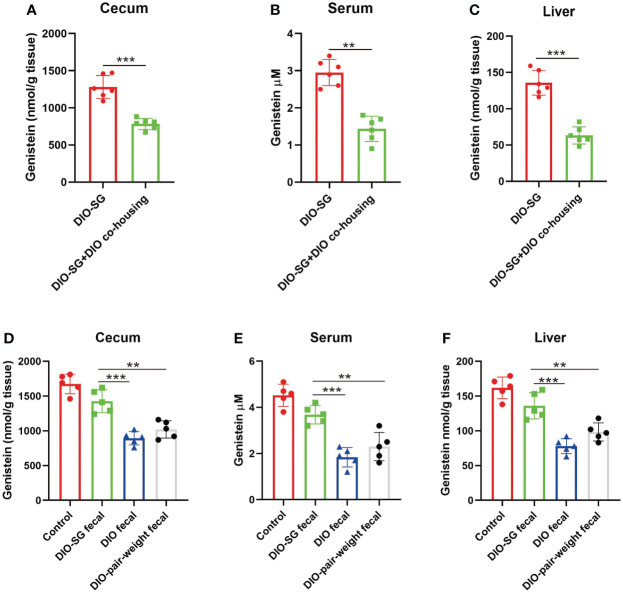
Transplantation of feces from SG mice could induce GE abundance in recipient mice. **(A–C)** Genistein concentrations in cecum, serum, and liver in different groups. n = 6. DIO-SG group: DIO mice underwent SG and fed alone in one cage. DIO-SG+DIO cohousing group: DIO mice underwent SG and cohoused with DIO mice (3 + 3 in one cage) for 6 weeks. **(D–F)** Genistein concentrations in cecum, serum, and liver in different groups. n = 5. There are four groups, in each group the normal-diet-fed mice are recipient mice and received oral gavage of feces from SG mice (DIO-SG fecal group), DIO mice (DIO fecal group), and pair-weight mice (DIO-pair-weight fecal group). The control group contains five mice which were fed with a normal diet and did not receive oral gavage. ANOVA test was used to compare the differences among the groups. Mann–Whitney U test was used to analyze the differences between the two groups. Data are presented as mean ± SEM, ** stands for p < 0.01, and *** stands for p < 0.001.

Since there is coprophage in mice, cohousing experiments were used to change the gut microbiota (22). The mice that underwent SG were cohoused with DIO mice. We found that the cohoused mice had decreased levels of GE (**Figures 6A–C**). We then used oral gavage to repeat the experiments. We directed 6-week-old normal mice with feces from SG mice, obese mice, and pair-weight control mice. Only feces from the SG mice could increase GE levels in the recipient mice (**Figures 6D–F**).

## Discussion

In this study, we found that in patients with obesity, the degree of NAFLD is negatively related to plasma GE abundance. In rodent models, diet-induced obese (DIO) mice also had decreased levels of genistein in the gut, liver, and serum. Moreover, the GE levels were restored after SG. Both *in vivo* and *in vitro* experiments showed that higher levels of GE could improve NAFLD in mice. Together, our finding suggested that GE has a therapeutic effect on NAFLD.

NAFLD is a progressive disease. The excessive accumulation of lipids constitutes the first stage (2). The more complex stage is NASH, characterized by the presence of hepatic steatosis and hepatocellular damage. The third stage is cirrhosis, HCC, and liver failure (23). Inflammatory processes are the key factors during the progression (1). A vast network of immune cells is mobilized during NAFLD/NASH, and numerous studies investigated the detailed mechanisms (1, 23). Previous studies reported that gut microbiota could cause inflammation in the liver (24). Interestingly, gavage with the “beneficial microbiota” could protect the mice against inflammation (25). It seems that certain gut microbiota had an anti-inflammation effect. Some studies showed that the gut microbiota could affect the function of intestinal epithelial cells (26). However, this could not explain the changes on the liver since the bacterium did not directly contact with the liver cells. On the other hand, gut microbiota has great potential to produce bioactive compounds such as metabolites (27). Our previous study showed that after SG, the metabolites may enter the liver *via* the intestinal vessels and then induce metabolic processes in the liver (10, 11). Thus, we further investigated the effects of metabolites on liver after SG.

We first screened the potential candidates. Blood samples from patients who received SG before and 3 months after SG were collected and analyzed by metabolite sequencing. Numerous metabolites were increased after surgery. Among these metabolites, we noticed a special candidate, genistein (GE). GE is abundant in leguminous plant food such as soybean and chickpeas (28). In the liver, GE is a potent phytoestrogen and could remit NASH through anti-lipid accumulation, antioxidation, and anti-apoptosis properties (29, 30). Thus, we speculated that increased postsurgical levels of GE could relieve NAFLD in patients with obesity.

A previous study showed that GE could protect the incubated human hepatocytes against NAFLD-like medium *via* the ER and GPER pathways (29). However, the role for GE after SG is unknown. To verify the effect of GE on the liver, we fed mice with water containing GE and took the liver for pathological examination. By HE staining, we found that after SG, there are fewer inflammatory cells infiltrated in the liver. This is further confirmed by an immunohistochemical study. There were a decreased number of macrophages (F4/80 +) in the liver after surgery. The tissue-resident phagocytes and the recruited macrophages could secrete cytokines and chemokines and contribute to NASH (31). Among the inflammatory molecules upregulated in NAFLD, monocyte chemoattractant protein-1 (MCP-1) is a special chemokine. It could promote migration of inflammatory cells by chemotaxis and integrin activation (32). It is positively correlated with obesity and NAFLD (33). In our study, we found that there are higher levels of MCP-1 in DIO mice. Supplement of GE could decrease MCP-1 levels. We also cultured macrophages *in vitro* and stimulated the cells with GE. Decreased levels of MCP-1 were also observed. Together, these findings identified that GE could contribute the restriction of cell infiltration.

IL-1β and TNF-α are important pro-inflammatory cytokines. They were markedly increased in NASH patients (34). A previous study suggested that chronic IL-1β and TNF-α exposure may activate specific populations of inflammatory cells in the liver (35). This further leads to NF-kB activation. Continuous NF-kB activation further results in downstream effects such as fibrosis, autophagy, and release of the reactive oxide species (ROS) (36). These are all important factors related to the progress of NAFDL to NASH. In our study, we found that GE could decrease the levels of IL-1β and TNF-α both *in vivo* and *in vitro*. By adding higher concentrations of GE to the cell culture system, there is less BMDM migration. These experiments suggested that GE may restrict cell migration by inhibiting IL-1β and TNF-α expression levels.

Furthermore, we investigated the mechanism of how SG induced the abundance of GE. Our study showed that in healthy mice there were higher levels of GE and in obese mice they were decreased. Moreover, the GE level was increased after SG in patients and in rodent models. Since we did not offer higher consumption of soybean-related food to patients after surgery, we wondered whether this change was caused by alteration of the gut microbiota. Coprophagy of mice inspired researchers to use cohoused mice to homogenize the gut microbiota (37). Since GE is a metabolite from the gut microbiota, it could be caused by changes of the gut microbiota after surgery. Then, what if mice underwent SG but their gut microbiota remained unchanged? It is very hard to fulfill this. However, by cohousing with obese mice, the SG mice could keep their gut microbiota nearly unchanged after surgery (**Supplementary Figure 1A**). We found that these mice did not have increased GE levels. This study showed that the gut microbiota is the key for the GE changes after SG.

It has already been fully verified that SG could significantly improve the metabolic state *via* weight-loss-dependent and independent mechanisms (38). On the one hand, rapid weight loss could largely remit the metabolic disorder. On the other hand, there are other reports showing that SG improved glycemia independent of weight loss by restoring hepatic insulin sensitivity and remodeling adipose tissue (39, 40). In our study, we tried to figure out whether the increase of plasma GE is caused by weight loss or surgery. The pair-weight control group was studied. In this group, mice received limited food to keep their weight at the same level to the SG group. Out study suggested that SG but not energy-restriction methods increased plasma GE. This finding suggested that SG increase GE *via* a weight-independent mechanism.

Finally, we tried to study whether additional supply of GE could enhance the NAFLD improvement after SG. Mice in the SG group was fed with water containing GE for 4 weeks, and we found a better remission of NAFLD. This finding showed that dietary supplement after bariatric surgery is very important. There are cases in which patients did not achieve satisfying weight loss and metabolic improvement. In addition to the surgical technique, nutrition management is also very important.

Together, our study identified genistein as a beneficial metabolite induced by SG. Genistein supplementation after SG could better alleviate liver inflammation and relieve NAFLD. Our study stressed the importance of dietary management after surgery. Surgery plus nutrient therapy could be a better treatment for patients with metabolic syndrome.

## Data availability statement

The datasets presented in this study can be found in online repositories. The names of the repository/repositories and accession number(s) can be found in the article/**Supplementary Material**.

## Ethics statement

The studies involving human participants were reviewed and approved by Ethics Committee of Wuhan Union Hospital. The patients/participants provided their written informed consent to participate in this study. The animal study was reviewed and approved by Animal Care Committee of Wuhan Union Hospital.

## Author contributions

Study design: GW and ZX. Animal experiments and mechanism study: YW and RZ. Clinical sample: JB, GL and YL. Formal analysis: SD and KT. Writing, reviewing, and editing: YW and GW. Performing surgery and supervising: ZX. Funding acquisition: GW. All authors contributed to the article and approved the submission.

## Supplementary material

Supplementary Figure 1 Quality control of the FMT experiments. **(A)** Principal component analysis (PCA) of gut microbiota before and after co-housing experiments. PCoA plots show significant differences in community from different groups in the gut. DIO-SG: The mice which had underwent SG. DIO: The diet induce obese mice. DIO-SG+DIO cohousing: SG mice cohoused with obese mice. n=5 in each group. The mice were female and age matched. **(B)** PCA of gut microbiota before and after oral gavage of feces experiments. PCoA plots show significant differences in community from different groups in the gut. **(C)** Serum GE levels in mice fed with HFD and HFD+GE. n=5.
