# Supplementary figures and images for: Increased plasma genistein after bariatric surgery could promote remission of NAFLD in patients with obesity
Source: Front Endocrinol (Lausanne). 2023 Jan 4;13:1024769. doi: 10.3389/fendo.2022.1024769 (PMC9846086; doi:10.3389/fendo.2022.1024769)

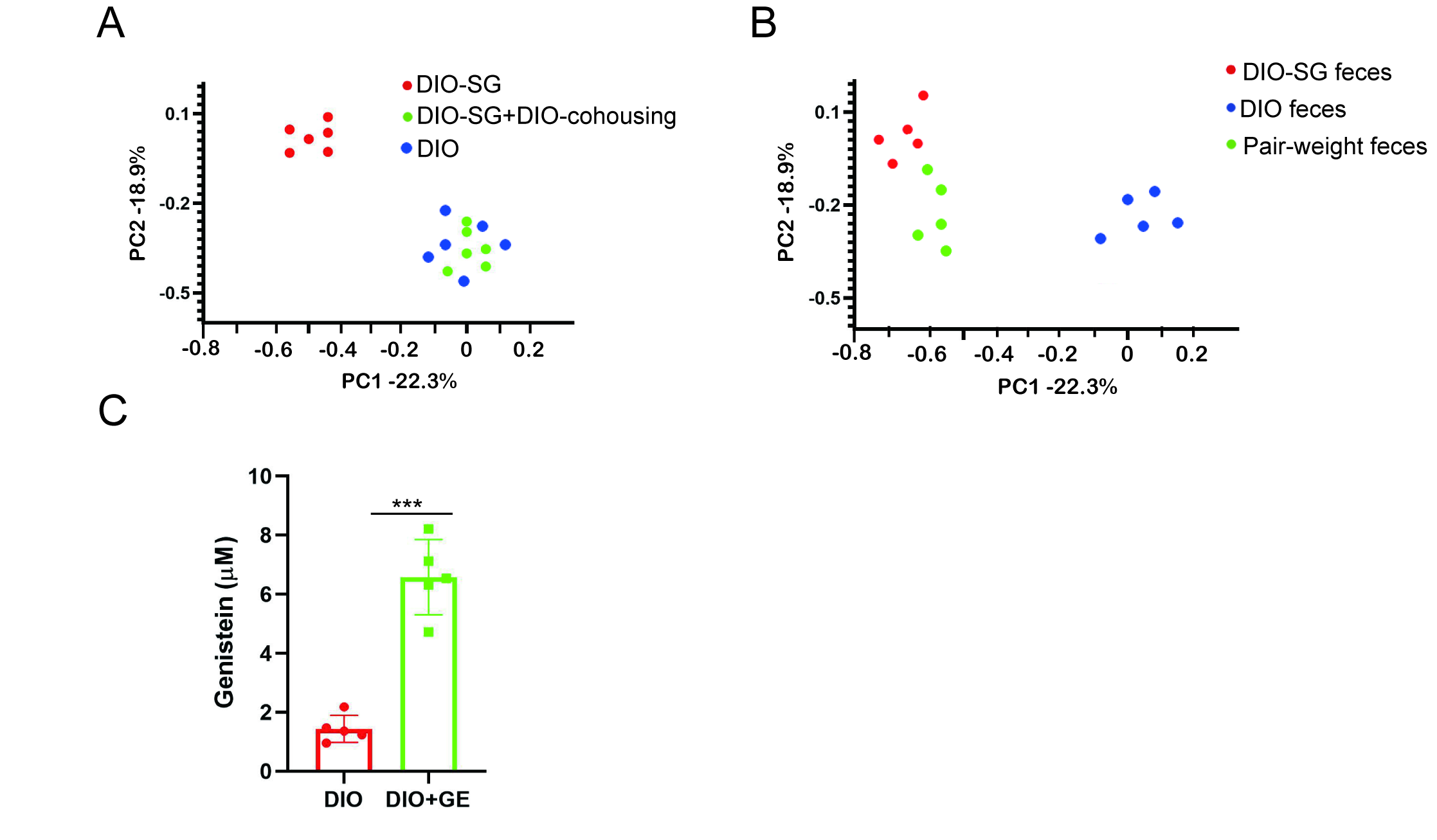

Supplement: Supplementary Figure 1 — Quality control of the FMT experiments. (A) Principal component analysis (PCA) of gut microbiota before and after co-housing experiments. PCoA plots show significant differences in community from different groups in the gut. DIO-SG: The mice which had underwent SG. DIO: The diet induce obese mice. DIO-SG+DIO cohousing: SG mice cohoused with obese mice. n=5 in each group. The mice were female and age matched. (B) PCA of gut microbiota before and after oral gavage of feces experiments. PCoA plots show significant differences in community from different groups in the gut. (C) Serum GE levels in mice fed with HFD and HFD+GE. n=5. [file Image_1.tif]
