# Supplementary material for: Increased plasma genistein after bariatric surgery could promote remission of NAFLD in patients with obesity
Source: Front Endocrinol (Lausanne). 2023 Jan 4;13:1024769. doi: 10.3389/fendo.2022.1024769 (PMC9846086; doi:10.3389/fendo.2022.1024769)
Supplement: Supplementary file 2 [file Table_1.docx]

**Table 1 Clinical baselines and characteristics before and 3 months after SG**

| Patient  number | 1 | 2 | 3 | 4 | 5 |
| --- | --- | --- | --- | --- | --- |
| Age | 32 | 23 | 31 | 35 | 27 |
| Gender | female | female | female | female | female |
| Baseline BMI | 40.6 | 43.7 | 36.4 | 38.1 | 44.1 |
| Post-surgical BMI | 31.7 | 34.2 | 26.5 | 29.1 | 36.4 |
| Baseline HbA1c | 8.3 | 7.6 | 7.8 | 6.0 | 6.5 |
| Type 2 DM | No | No | No | No | No |
| Hypertension | No | No | No | Yes | No |
| NAFLD | Yes | Yes | Yes | Yes | Yes |
| Baseline ALT  (U/L) | 189 | 153 | 167 | 59 | 172 |
| Post-surgical ALT (U/L) | 87 | 69 | 64 | 53 | 102 |
| Baseline AST  (U/L) | 78 | 45 | 63 | 45 | 83 |
| Post-surgical AST (U/L) | 36 | 36 | 35 | 32 | 70 |
| Baseline UA (μmol/L) | 530 | 438 | 462 | 403 | 515 |
| Post-surgical UA (μmol/L) | 512 | 422 | 477 | 371 | 428 |
| Baseline LDL(mmol/L) | 2.97 | 3.53 | 5.53 | 2.79 | 4.26 |
| Post-surgical LDL(mmol/L) | 2.72 | 3.26 | 3.97 | 2.53 | 3.45 |
| Baseline HDL  (mmol/L) | 0.71 | 0.84 | 0.75 | 1.04 | 0.67 |
| Post-surgical  HDL(mmol/L) | 0.89 | 1.13 | 1.01 | 1.27 | 0.94 |
