# Supplementary material for: Increased plasma genistein after bariatric surgery could promote remission of NAFLD in patients with obesity
Source: Front Endocrinol (Lausanne). 2023 Jan 4;13:1024769. doi: 10.3389/fendo.2022.1024769 (PMC9846086; doi:10.3389/fendo.2022.1024769)
Supplement: Supplementary file 3 [file Table_2.docx]

| Gene name | Primers |
| --- | --- |
| *Tnfa*-F | CTGGATGTCAATCAACAATGGGA |
| *Tnfa*-R | ACTAGGGTGTGAGTGTTTTCTGT |
| *IL-1β*-F | GAAATGCCACCTTTTGACAGTG |
| *IL-1β*-R | TGGATGCTCTCATCAGGACAG |
| *Mcp-1*-F | TTAAAAACCTGGATCGGAACCAA |
| *Mcp-1*-R | GCATTAGCTTCAGATTTACGGGT |
| *Gapdh*-F | AGGTCGGTGTGAACGGATTTG |
| *Gapdh*-R | TGTAGACCATGTAGTTGAGGTCA |
